# Exploring the singularity of human neurons: keep calm and carry on

**DOI:** 10.3389/fnsyn.2025.1672646

**Published:** 2025-10-02

**Authors:** Baptiste Libé-Philippot

**Affiliations:** Aix-Marseille Université, CNRS, Developmental Biology Institute of Marseille (IBDM), NeuroMarseille, Marseille, France

**Keywords:** human brain evolution, neuronal development and maturation, human gene duplicates, synaptic neoteny, neuronal excitability, cerebral cortex, *ex vivo* brain sections, neurodevelopmental disorders

## Abstract

The human brain’s increased cognitive abilities are underpinned by evolutionary adaptations at the molecular, cellular, and circuit levels of neural structures. This perspective explores how protracted neuronal development and divergent cell intrinsic neuronal properties, including neuronal excitability, contribute to human neurobiological singularity. Those cellular aspects rely on molecular evolutionary innovations, including evolution of gene regulation and gene duplications that play critical roles in prolonging synaptogenesis and reducing neuronal excitability. These molecular evolutionary innovations are shown to interact with core neurodevelopmental molecular pathways linked to neurodevelopmental disorders. Furthermore, complementary multimodal and multiscale approaches offer promising platforms to study these processes and develop species-relevant therapeutic strategies. They include *ex vivo* acute brain slices and organotypic cultures which offer emerging tools for understanding human species-specificities and neural disorders.

## Introduction

Near my laboratory, located in the Calanques of Marseille (France), lies the underwater Cosquer Cave. Within its submerged depths, prehistoric paintings created between 27,000 and 14,000 BC provide a striking glimpse into the distant past of *Homo sapiens*. The artwork depicts various animals—horses, ibex, deer, bison, aurochs, seals, and penguins—as well as human symbols, including genital representations and stencils of human hands (Clottes et al., 1992). While the precise meaning of these anthropological signs remains elusive, they undoubtedly represent the cognitive and cultural evolution that distinguishes *Homo sapiens* from other primate, hominid and archaic hominin species. Notably, these features, including abstract thinking, cultural transmission, social learning, cooperation, and language (Richerson et al., 2021; Sherwood and Gómez-Robles, 2017; Lancaster, 2024; Zeberg et al., 2024), are underpinned by neurobiological substrates that evolved alongside morphological, metabolic, and immune system changes (Zeberg et al., 2024; Pollen et al., 2023).

**Figure 1:**
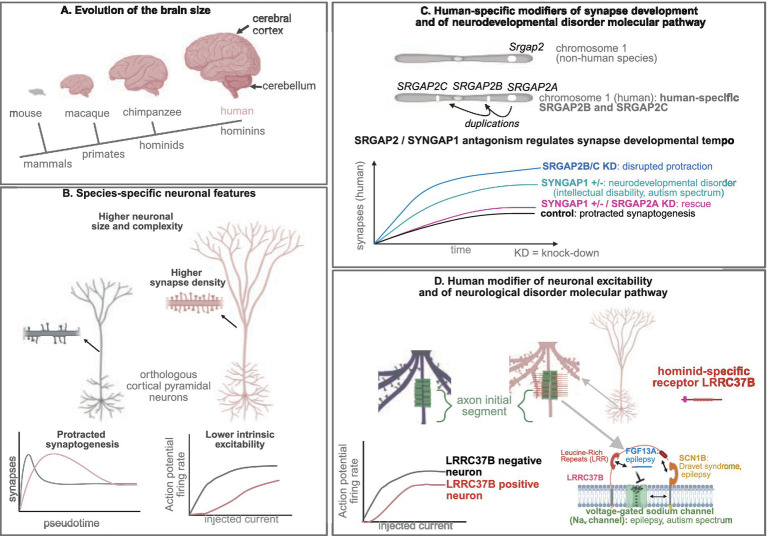
Human modifiers of neuronal development and physiology modulate neuronal disease pathways. **(A)** Evolution of the brain size. **(B)** Species-specific neuronal features. **(C)** Human-specific modifiers of synapse development and of neurodevelopmental disorder molecular pathway. **(D)** Human modifier of neuronal excitability and of neurological disorder molecular pathway.

What is the biological substrate responsible for such evolutionary advancements? Over the past four decades, research has pointed to the increased size of the human brain, particularly the cerebral cortex—the outermost layer of the brain involved in sensory processing and higher cognitive functions—as a central element in the evolution of human cognition (Figure 1A). This expansion has been associated with a larger number of neurons and more complex cytoarchitecture within the cerebral cortex, which together contribute to the increased cognitive abilities of humans. These changes primarily result from the evolution of neurodevelopmental processes, especially those governing neural proliferation, neurogenesis, and fate determination during the prenatal period (Sherwood and Gómez-Robles, 2017; Lancaster, 2024; Libé-Philippot and Vanderhaeghen, 2021; Lindhout et al., 2024; Vanderhaeghen and Polleux, 2023; Kelley and Pașca, 2022; Namba and Huttner, 2024).

However, despite significant advances in our understanding of these developmental processes, relatively little is known about the evolution of the fundamental building blocks of the brain—the neurons themselves—and the circuits they form (Lancaster, 2024; Vanderhaeghen and Polleux, 2023; Libé-Philippot et al., 2024). Humans share most cortical neuron types with other mammalian and primate species, yet these neurons exhibit morphological and physiological differences that may be central to the evolution of human cognition. These differences are thought to be linked to species-specific gene expression patterns and human-specific modifiers of ancestral molecular mechanisms, including pathological ones (Lancaster, 2024; Pollen et al., 2023; Vanderhaeghen and Polleux, 2023; Libé-Philippot et al., 2024; Wallace and Pollen, 2024).

## Carry on slowly: protracted synaptogenesis and enhanced learning abilities

A particularly striking feature of human neurodevelopment is the protracted pace of neuronal development. This phenomenon, known as heterochrony, bradychrony, or neoteny, refers to the delayed maturation of key neurodevelopmental processes, including corticogenesis and synaptic maturation, in humans compared to other primate species (Sherwood and Gómez-Robles, 2017; Lancaster, 2024; Libé-Philippot and Vanderhaeghen, 2021; Lindhout et al., 2024; Petanjek et al., 2011; Zhou et al., 2024; McNamara, 2012). Notably, neoteny of the synaptogenesis—the process through which neurons form connections in a highly plastic manner (Sherwood and Gómez-Robles, 2017; Waites et al., 2005)—is thought to be the foundation for the enhanced learning abilities characteristic of *Homo sapiens* (Gould, 1992; Bufill et al., 2011). Each step of this protracted neurodevelopment could follow different modalities of heterochrony, influenced by various mechanisms, including epigenetic regulation, metabolic processes, protein targeting to synapses, and human-specific modifiers that regulate these processes (Libé-Philippot and Vanderhaeghen, 2021; Casimir et al., 2024; Ciceri and Studer, 2024). For example, synaptogenesis takes approximately 5–10 years in humans, compared to months in macaques and weeks in mice, while corticogenesis lasts months in humans, as opposed to weeks in macaques and days in mice (Libé-Philippot and Vanderhaeghen, 2021; Lindhout et al., 2024; Libé-Philippot et al., 2024). Furthermore, it is conceivable that different brain regions undergo varying rates of developmental maturation, with synaptogenesis showing more pronounced heterochronicity in areas such as the prefrontal cortex—region associated with higher cognitive functions (Petanjek et al., 2011)—compared to primary sensory and motor areas (Sherwood and Gómez-Robles, 2017).

Understanding the molecular and cellular substrates underlying this protracted neurodevelopment is pivotal for uncovering the distinctive cognitive abilities of humans. It is also critical to understanding fundamental bases of neurodevelopmental disorders since they may be intimately linked to disturbed pace of synapse development, in particular in autism spectrum disorder and schizophrenia (Penzes et al., 2011). Previous studies have shown that neurons derived from human, chimpanzee and mouse pluripotent stem cells and xenotransplanted into mouse cerebral cortex, mature at their own pace (Libé-Philippot and Vanderhaeghen, 2021; Vanderhaeghen and Polleux, 2023; Linaro et al., 2019; Espuny-Camacho et al., 2013; Marchetto et al., 2019; Gaspard et al., 2008). This suggests that the pace of neuronal development is primarily driven by cell-intrinsic, species-specific mechanisms, included at the synaptic maturation level (Libé-Philippot et al., 2024).

What molecular mechanisms underpin these changes in the pace of development? Many of the developmental processes, cell types, and gene expression patterns involved in neurodevelopment are highly conserved across vertebrate species, with basic neuronal and synaptic functions shared even among distant metazoan taxa (Lancaster, 2024; Libé-Philippot and Vanderhaeghen, 2021; Zhou et al., 2024; Tosches, 2021). However, many of the genomic innovations specific to the human lineage are linked to neurodevelopmental and neuronal physiological processes. On examples are mutations in cis-regulatory elements that represent about 1% of the genomic differences between *Homo sapiens* and chimpanzees and that result in novel gene expression patterns (Pollen et al., 2023; Libé-Philippot and Vanderhaeghen, 2021; Lindhout et al., 2024; Vanderhaeghen and Polleux, 2023; Kelley and Pașca, 2022; Libé-Philippot et al., 2024; Zhou et al., 2024; Whalen and Pollard, 2022; King and Wilson, 1975). This includes human gain enhancers in the more than 3,000 human accelerated regions, which are largely non-coding regulatory genomic regions, highly conserved between mammalian species but divergent in the human genome, that are particularly active in neural processes (Pollen et al., 2023; Libé-Philippot and Vanderhaeghen, 2021; Lindhout et al., 2024; Vanderhaeghen and Polleux, 2023; Kelley and Pașca, 2022; Libé-Philippot et al., 2024; Zhou et al., 2024).

These regulatory changes can lead to species-specific differential patterns of gene expression. For instance, *OSTN* (*osteocrin*) is a muscle and bone secreted protein but expressed in the brain only in primate species. It regulates the protracted maturation of the dendritic tree (Ataman et al., 2016). This could be explained by the presence in the genomes of primate species of binding sites to the transcription factors of the MEF2 family, involved in synaptic maturation (Ataman et al., 2016). Interestingly, *MEF2A* was identified to display a protracted developmental expression pattern in the human cerebral cortex compared to other primate species (Liu et al., 2012). Striking experimental works revealed human-specific deletions in cis-regulatory elements of *CBLN2* (*cerebellin 2*), and higher retinoic acid signaling in the primate prefrontal cortex, which led to *CBLN2* higher levels of expression leading to increase synapse formation and cortical connectivity (Shibata et al., 2021; Shibata et al., 2021).

Another level of molecular evolutionary novelties relies on segmental gene duplications, such as species-specific gene duplicates (Pollen et al., 2023; Libé-Philippot and Vanderhaeghen, 2021; Lindhout et al., 2024; Vanderhaeghen and Polleux, 2023; Kelley and Pașca, 2022; Libé-Philippot et al., 2024; Zhou et al., 2024; Bailey et al., 2002; Soto et al., 2025). One well-documented example of such a genomic innovation is the *SRGAP2* (*SLIT-ROBO Rho GTPase Activating Protein 2*) gene family, specifically the human-specific *SRGAP2B* and *SRGAP2C* genes. These genes, which arose during the emergence of *Homo* species, have been shown to *induce* protracted synaptic maturation and enhanced neuronal connectivity when overexpressed in mouse cortical neurons, leading to enhanced cortical connectivity and learning abilities (Lancaster, 2024; Pollen et al., 2023; Libé-Philippot and Vanderhaeghen, 2021; Vanderhaeghen and Polleux, 2023; Kelley and Pașca, 2022; Namba and Huttner, 2024; Charrier et al., 2012; Schmidt et al., 2021). It was recently confirmed that *SRGAP2B* and *SRGAP2C* are essential for protracted synaptic maturation, as demonstrated by the knockdown of their expression in human cortical neurons xenotransplanted into the mouse cerebral cortex (Figure 1C) (Libé-Philippot et al., 2024). Surprisingly, these experiments revealed that the acceleration of synaptic development was more pronounced than expected: at 18 months post-transplantation, the neurons had reached synaptic densities similar to those observed in 5–10-year-old children.

Furthermore, the experiments uncovered a novel molecular mechanism involving a competition between the synaptic proteins SRGAP2A and SYNGAP1 (Synaptic Ras GTPase-activating protein 1), which regulate the timing of synaptogenesis in mammals, with SRGAP2B and SRGAP2C acting as human-specific modifiers (Libé-Philippot et al., 2024). *SYNGAP1* is a major gene responsible for intellectual disability and autism spectrum disorder (Gamache et al., 2020). One cellular phenotype of *SYNGAP1* haploinsufficiency is a precocious synaptic development or disrupted neoteny (Vermaercke et al., 2024), as observed in some forms on autism spectrum disorder (Penzes et al., 2011). Interestingly, SYNGAP1 postsynaptic synaptic accumulation and the phenotype of accelerated synaptogenesis could be rescued while performing *SRGAP2A* knock-down in a *SYNGAP1* haploinsufficiency genetic background (Libé-Philippot et al., 2024), opening possibilities for future therapeutic approaches (Figure 1C).

Those recent studies highlight several levels of breaks that act on the protraction of the synaptic development. While the evolution of gene regulation (e.g., epigenetics, non-coding genomic regions) acts on differences in the pattern and pace at the transcription step (Pollen et al., 2023; Libé-Philippot and Vanderhaeghen, 2021; Lindhout et al., 2024; Vanderhaeghen and Polleux, 2023; Kelley and Pașca, 2022; Libé-Philippot et al., 2024; Zhou et al., 2024; Ciceri and Studer, 2024; Ataman et al., 2016; Liu et al., 2012; Shibata et al., 2021; Shibata et al., 2021), and are easily assessed by transcriptomic studies, additional non-genetic post-transcriptional breaks act on the protein abundance at the synapse. Indeed, the developmental pace of variation in the synaptic abundance of some proteins does not fit with the variation of the transcripts, suggesting post-transcriptional and/or translational and/or protein stability regulation (Wang et al., 2023). Such regulation, involves the activity of small GTPases that could mediate synapse targeting (Wang et al., 2023) or the interaction between human-specific proteins with their ancestors that can lead to their degradation (Libé-Philippot et al., 2024; Assendorp et al., 2024). This indicates that future studies on (local) translation, protein stability, protein co-trafficking and cell-state dependant synaptic targeting, beyond cross-species transcriptional comparisons, could be beneficial for a deeper understanding on the evolution of synaptic development and structure.

One could wonder why several levels of mechanisms or breaks evolved in the same direction, e.g., a protracted neuronal and synaptic maturation. On one hand, this could ensure the robustness (Hiesinger and Hassan, 2018) of this key human developmental feature. On the other hand, a strong robustness to excessive variations, e.g., neotenic disruption, allows subtle variations (Hiesinger and Hassan, 2018). One could extrapolate that different scales of subtle variations could be key in the development of human circuits: (1) variations between neurons/synapses that may be crucial to achieve developmental robustness (Hiesinger and Hassan, 2018), (2) it could be involved in different paces of development between neuronal compartments (e.g., dissociate the pace of development of synapse subtypes, synapses versus axon, dendrites, etc.), or (3) between cerebral cortex area (e.g., a higher protraction in the prefrontal cortex versus motor cortex). Moreover, one could imagine that such profusion of breaks could have participated in the evolvability of *Homo* species.

## Keep calm: reduced neuronal excitability and enhanced computational properties

While much attention has been paid to the developmental aspects of human neuronal evolution, less is known about the species-specific physiological characteristics of human cortical neurons and the underlying mechanisms that give rise to these traits (Vanderhaeghen and Polleux, 2023; Libé-Philippot et al., 2024). Cross-species comparisons from *ex vivo* brain sections, originating from non-pathological surgical resections provided human specificities at the morphological, physiological and connectivity levels, in the cerebral cortex (pyramidal excitatory neurons and GABAergic interneurons), in the hippocampus (connectivity) and in the cerebellum (Purkinje cells) (Libé-Philippot et al., 2023; Beaulieu-Laroche et al., 2021; Beaulieu-Laroche et al., 2018; Kalmbach et al., 2018; Wilson et al., 2025; Watson et al., 2025; Mohan et al., 2015; Eyal et al., 2014; Deitcher et al., 2017; Hunt et al., 2023; Chartrand et al., 2023; Busch and Hansel, 2023; Masoli et al., 2024; Campagnola et al., 2022; Szegedi et al., 2020; Molnár et al., 2016; Wilson et al., 2025; Oláh et al., 2025; Csemer et al., 2023; Kalmbach et al., 2021; Wilbers et al., 2023), suggesting co-evolution of brain regions, for which remains the question of the underlying mechanisms (common molecular innovations, adaptation, etc.). Morphologically, human neurons are larger, exhibiting more elaborate dendritic arborization and a greater number of synapses leading to higher neural connectivity compared to other primates. These features are thought to contribute to the enhanced computational properties of human neurons (Vanderhaeghen and Polleux, 2023; Libé-Philippot et al., 2024). At the electrophysiological level, human neurons are more compartmentalized, less excitable, and capable of generating long trains of action potentials when engaged in cognitive tasks, compared to neurons in other mammals and primates (Figure 1B) (Vanderhaeghen and Polleux, 2023; Libé-Philippot et al., 2024). Fine tuning of intrinsic neuronal excitability is critical since disturbed intrinsic neuronal excitability is intimately linked to neurological disorders, including epilepsy, migraine and neurodegenerative disorders (Wijesinghe and Camp, 2011). More attention will be probably paid in the coming years at the scales beyond neuronal properties, for instance circuit structure and computational properties.

Those divergent cellular features should rely on molecular novelties, including divergence in cis-regulatory elements, even though a comprehensive molecular substrate for those evolutionary divergent features is far to be understood. These changes can lead to differential patterns of gene expression. For instance genes differentially expressed in human cortical pyramidal neurons compared to other primate and hominid species are notably linked to synaptic compartments’ structure and physiology (Jorstad et al., 2023). Moreover, among human duplicated genes, *FRMPD2B* and *LRRC37B* should play a critical role in the divergence of human neurons, *FRMPD2B* in synaptic signaling (Soto et al., 2025) and *LRRC37B* in neuronal excitability (Libé-Philippot et al., 2023).

It was recently shown that human cortical neurons exhibit greater diversity in the excitability of their axon initial segment (AIS), the subcellular compartment where action potentials are initiated (Libé-Philippot et al., 2023). The consequence at the circuit and information processing levels remains to be explored. This lower excitability could be an adaptive response to the increased neural connectivity in humans, resulting in higher accuracy of information processing. This could contribute to allowing for sustained trains of action potentials during cognitive tasks without compromising signal fidelity. This altered excitability may also modulate information processing at both the neuronal and circuit levels by influencing neuronal gain and increasing neuronal diversity (Libé-Philippot et al., 2024).

A pivotal discovery was the identification of the hominid-specific transmembrane protein LRRC37B (Leucine Rich Repeat Containing 37B), which is localized to the AIS of a subset of human cortical neurons. Strikingly, LRRC37B was found to reduce neuronal excitability at the level of the AIS (Libé-Philippot et al., 2023). Interestingly, humans possess more than 15 paralogs of the *LRRC37* gene family, which encodes transmembrane proteins with leucine-rich extracellular domains. Among these paralogs, *LRRC37B* is specific to humans and hominids (including chimpanzees), differing from the other paralogs and the ancestral *Lrrc37a* found in other amniotes (Libé-Philippot et al., 2023; Giannuzzi et al., 2013). The ancestral *Lrrc37a* gene is not expressed in the mouse cerebral cortex and *LRRC37B* transcript is expressed at higher levels in human cortical pyramidal neurons than in chimpanzees (Libé-Philippot et al., 2023). Moreover, the LRRC37B protein is not detected at the AIS of the chimpanzee cortical pyramidal neurons (Figure 1D) (Libé-Philippot et al., 2023).

The AIS, a crucial site enriched with voltage-gated sodium channels (Na_v_), which are essential for action potential generation (Libé-Philippot et al., 2023). Using various experimental approaches, it was demonstrated that LRRC37B interacts with two key modulators of Na_v_ channels—secreted FGF13 (fibroblast growth factor 13) isoform A (FGF13A) and the transmembrane protein SCN1B (β-subunit of Na_v_)—to modulate neuronal excitability (Libé-Philippot et al., 2023). *Ex vivo* electrophysiological recordings showed that LRRC37B overexpression in mouse cortical pyramidal neurons enhances the inhibitory effect of FGF13A on Na_v_ channels, thus decreasing neuronal excitability at the AIS (Figure 1D) (Libé-Philippot et al., 2023). One could imagine that exploring how to modulate the LRRC37B–FGF13A–SCN8A interaction to act on neuronal excitability, could be useful to cure epileptic disorders.

Therefore, LRRC37B is a human species-specific modulator of AIS and neuronal excitability and it acts by concentrating FGF13A function on Na_v_ channels (Figure 1D) (Libé-Philippot et al., 2024; Libé-Philippot et al., 2023). This work opens an avenue to address many questions in the future, including the consequences of a diverse lower excitability of the AIS to the neural circuit function and information processing.

Another aspect that this study highlight is the heterogeneity in the protein composition of a neuronal compartment, i.e., a subpopulation of any neuronal subtype express LRRC37B protein at their AIS (Libé-Philippot et al., 2023), between cortical neurons. This follows other studies showing a higher diversity or specialization of neurons in the human cerebral cortex compared to other species (Jorstad et al., 2023; Berg et al., 2021). This challenges the definition of neuronal type defined by transcript marker expression, as recently done in the zebrafish in which transcriptionally similar neurons can be functionally diverse (Shainer et al., 2025). One could wonder which transcriptional and post-transcriptional mechanisms evolved in humans leading to potential higher molecular diversity within neuronal populations. This can involve for instance cell-state dependent mechanisms, translational or sub-compartment protein targeting mechanisms, morphological/synaptic innervation dependent mechanisms, to be studied further. Regarding the potential impact of such higher diversity or specialization, one could wonder whether this led to changes in neural processing, including the reliability and robustness of neural information processing, functional specialization, complexity of neuronal information transmission, robust learning (Wu et al., 2025; Perez-Nieves et al., 2021; Gjorgjieva et al., 2016). Novel experimental models and computational biology should help in the near future to elucidate which of those functional properties could have emerged from the human neuronal evolution, beyond expanded cortical size.

## Species-specific sensitivities to neurodevelopmental and brain disorders

Interestingly, many of the cellular processes involved in human neural evolution and the genes that distinguish the human lineage are closely tied to neurodevelopmental disorders, aging, and brain diseases (Libé-Philippot and Vanderhaeghen, 2021; Zhou et al., 2024; Vickery et al., 2024; Douaud et al., 2014). For instance, dysregulation of neurodevelopmental processes, such as those occurring during neural proliferation and that evolved in humans, can lead to defects in the final brain cytoarchitecture, resulting in conditions such as microcephaly and macrocephaly (Libé-Philippot and Vanderhaeghen, 2021). Mutations in human accelerated regions, while divergent to all other mammalian species, are enriched in individuals with neurodevelopmental disorders and underlie for instance 5% of consanguineous cases of autism spectrum disorders (Doan et al., 2016). For instance, *MEF2* genes and their binding sites are linked to autism spectrum disorder (Chaudhary et al., 2021). Some other genes displaying human species-specific developmental patterns of expression like *CBLN2* code for proteins that are ligands to receptors tightly linked to neurodevelopmental disorders (e.g., neurexins) (Südhof, 2023), suggesting that they could mediate species-specific sensitivities to those disorders.

Some human duplicated genes reside in genomic hotspots linked to neurodevelopmental disorders, including autism spectrum disorder (Soto et al., 2025). Other human duplicated genes, and notably *SRGAP2C* and *LRRC37B*, are loss-of-function intolerant, suggesting strong levels of purifying selection (Soto et al., 2025). They could act as species-specific modifiers of molecular pathways implicated in neurodevelopmental disorders (Libé-Philippot et al., 2024; Assendorp et al., 2024). Specifically, the *SRGAP2* gene family, and particularly the human-specific genes *SRGAP2B* and *SRGAP2C*, were functionally linked to SYNGAP1 and CTNND2, two synaptic proteins associated with intellectual disabilities, autism spectrum disorders, and Cri-du-Chat syndrome (Libé-Philippot et al., 2024; Assendorp et al., 2024). Additionally, the hominid-specific protein LRRC37B was demonstrated to interact with FGF13A, SCN1B and SCN8A that are involved in epilepsy, Dravet syndrome, and autism spectrum disorder (Libé-Philippot et al., 2023). These results highlight the possibility of species-specific sensitivities to neurodevelopmental and neurological disorders that that can be critical in the diagnosis, patient management and therapeutical approaches.

In our knowledge, neurodevelopmental disorders like autism spectrum and schizophrenia have not been described in nonhuman primates, even though common genetic and social behavioral traits have been identified compared to other species, including chimpanzees (Li et al., 2021; Faughn et al., 2015; Yoshida et al., 2016; Crow, 1997). It would mean that such disorders are the consequence of genomic trade-offs between neural circuit evolution and harmful effects in the variation of their development and structure (Sikela and Searles Quick, 2018). This apparent evolution of vulnerability to neurodevelopmental disorders could result from human species-specific causes of such disorders (e.g., genetic evolution and protracted development), natural selection in nonhuman species against such variations, or on the fact that the definition of such disorders are based on behavioral traits expanded in humans (e.g., language). Moreover, humans display species-specific gray matter decline linked to aging in cerebral cortex area that diverged in size compared to chimpanzees (notably, the prefrontal and frontal cerebral cortex) (Vickery et al., 2024). While several animal species, including non-human primates, display age-related amyloid-β and tau accumulation, there is debate on whether cellular loss and behavioral disorders linked to Alzheimer’s disease might be a human-specific disorder (Devinsky et al., 2018; Finch and Austad, 2015).

## Human *ex vivo* approaches for basic research and drug development

**Figure 2:**
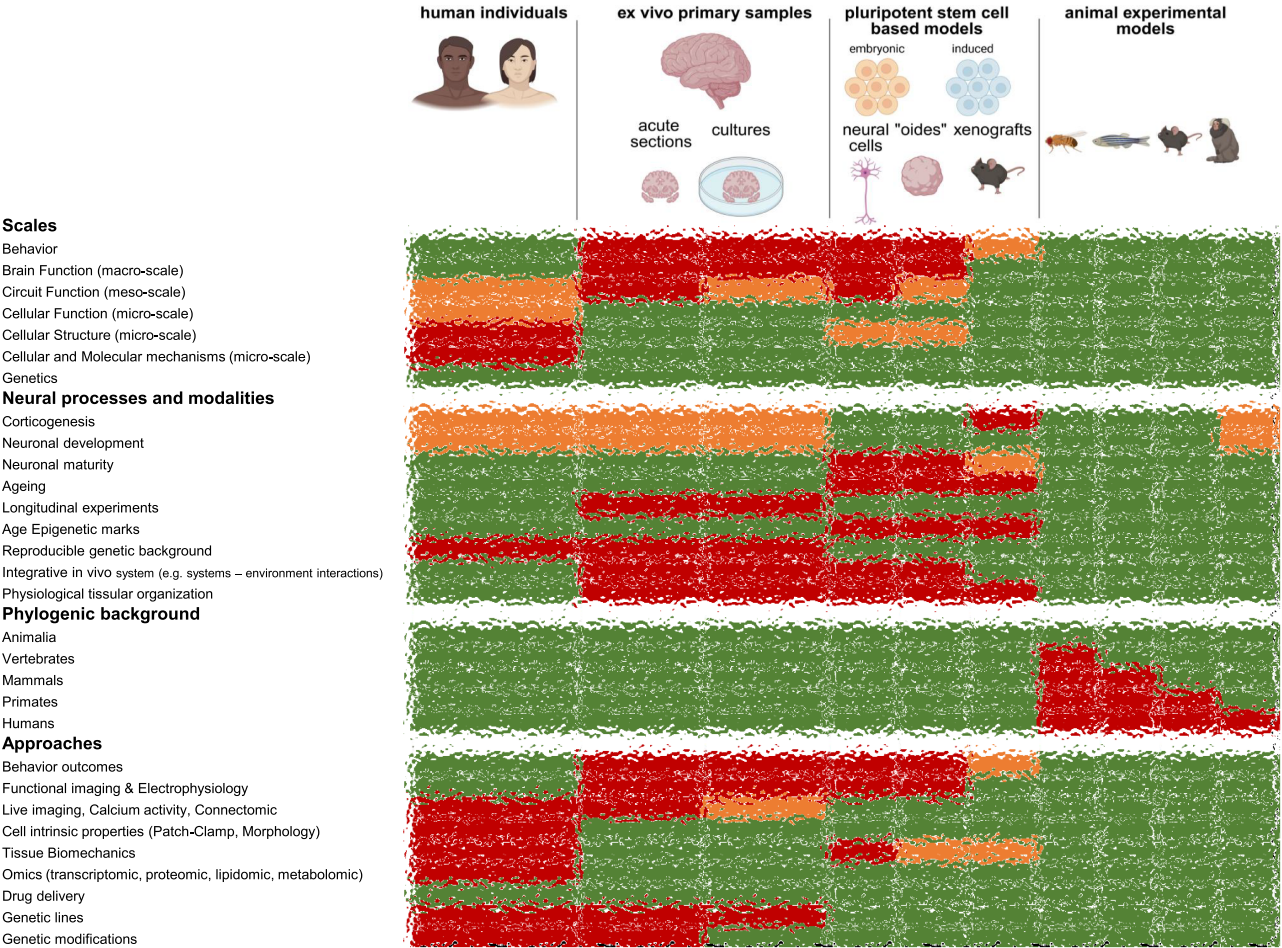
Experimental models to study human brain development, aging, disorders and evolution. In green accessible aspects of the models, in orange limited access, in green accessible aspects.

How studying human brain basic development, function, and disorders? Answering this question necessitates multimodal and multiscale approaches. Indeed, depending on the scale of the study, from genes to cell to circuit to behavior, one could consider human individuals themselves, primary samples, human pluripotent stem cell-based models (2D differentiations, organoids, assembloids) or animal experimentation (Figure 2). These approaches differ not only on the accessible scales but also on the stages and neural processes they can address. Moreover, every biological technology and approach cannot be accessible by each of those approaches on its own.

In this context, spare human brain tissues obtained from neurosurgical procedures can be used in acute conditions to address cellular properties, morphologies, “omic” (genomic, epigenetic, transcriptomic, proteomic, metabolomic, lipidomic, etc.) and molecular questions (Libé-Philippot et al., 2023; Beaulieu-Laroche et al., 2021; Beaulieu-Laroche et al., 2018; Kalmbach et al., 2018; Wilson et al., 2025; Watson et al., 2025; Mohan et al., 2015; Eyal et al., 2014; Deitcher et al., 2017; Hunt et al., 2023; Chartrand et al., 2023; Busch and Hansel, 2023; Masoli et al., 2024; Campagnola et al., 2022; Szegedi et al., 2020; Molnár et al., 2016; Wilson et al., 2025; Oláh et al., 2025; Csemer et al., 2023; Kalmbach et al., 2021; Wilbers et al., 2023; Wilbers et al., 2023; Kerkhofs et al., 2018; Wierda et al., 2024; Lee et al., 2023; Kim et al., 2023; Bernard et al., 2004; Buchin et al., 2022; Ting et al., 2018; Gidon et al., 2020; Lee et al., 2023; Szegedi et al., 2024; Bocchio et al., 2019; Szegedi et al., 2023; Szegedi et al., 2017; Yang et al., 2025; Yang et al., 2024; Szegedi et al., 2016; Barzó et al., 2025; Guet-McCreight et al., 2023; Rich et al., 2022; Mertens et al., 2024; Moradi Chameh et al., 2021; Goriounova et al., 2018). Interestingly, neuronal physiology can be combined with *post-hoc* morphological and connectivity reconstructions, protein immunostaining and transcriptomic approaches. Such approaches can be of interest to address questions on mature and aging tissues because of the protracted neuronal development that make other human models’ immature, and on a genetic and epigenetic human and aged background (Figure 2). The sections come from cortical and hippocampal regions, mostly, and from drug-resistant or cancer patients. Importantly, fresh autopsies could be an alternative source of tissue, with control conditions and offering more diverse brain regions (Verwer et al., 2002; Verwer et al., 2002; Plug et al., 2024). This enabled to study non-pathological regions (e.g., tissues with no lesions surrounding an epileptic focus or a tumor) (Libé-Philippot et al., 2023; Beaulieu-Laroche et al., 2021; Beaulieu-Laroche et al., 2018; Kalmbach et al., 2018; Wilson et al., 2025; Watson et al., 2025; Mohan et al., 2015; Eyal et al., 2014; Deitcher et al., 2017; Hunt et al., 2023; Chartrand et al., 2023; Busch and Hansel, 2023; Masoli et al., 2024; Campagnola et al., 2022; Szegedi et al., 2020; Molnár et al., 2016; Wilson et al., 2025; Oláh et al., 2025; Csemer et al., 2023; Kalmbach et al., 2021; Wilbers et al., 2023; Wilbers et al., 2023; Kerkhofs et al., 2018; Wierda et al., 2024; Lee et al., 2023; Kim et al., 2023; Ting et al., 2018; Gidon et al., 2020; Lee et al., 2023; Szegedi et al., 2024; Bocchio et al., 2019; Szegedi et al., 2023; Szegedi et al., 2017; Yang et al., 2025; Yang et al., 2024; Szegedi et al., 2016; Barzó et al., 2025; Guet-McCreight et al., 2023; Mertens et al., 2024; Moradi Chameh et al., 2021; Goriounova et al., 2018), pathological regions (e.g., epileptic focus) (Bernard et al., 2004; Buchin et al., 2022; Rich et al., 2022) and cross-species comparisons (e.g., rodents versus nonhuman primates versus humans) (Libé-Philippot et al., 2023; Beaulieu-Laroche et al., 2021; Beaulieu-Laroche et al., 2018; Kalmbach et al., 2018; Wilson et al., 2025; Watson et al., 2025; Mohan et al., 2015; Eyal et al., 2014; Deitcher et al., 2017; Hunt et al., 2023; Chartrand et al., 2023; Busch and Hansel, 2023; Masoli et al., 2024; Campagnola et al., 2022; Szegedi et al., 2020; Molnár et al., 2016; Wilson et al., 2025; Oláh et al., 2025; Csemer et al., 2023; Kalmbach et al., 2021; Wilbers et al., 2023).

Interestingly, those acute recordings enable to assess the acute effect of organic compounds (e.g., caffein, receptor agonists/modulator or channel blockers) (Kerkhofs et al., 2018; Bocchio et al., 2019; Szegedi et al., 2023; Yang et al., 2025; Yang et al., 2024), ideally in a dose–response manner, on electrophysiological properties. This is of particular interest for drugs that target proteins selectively expressed or higher expressed in humans, like HCN channels (Kalmbach et al., 2018; Szegedi et al., 2023), or for confirmation of results got in non-human animals (Yang et al., 2025).

Importantly, some studies described evolution of electrophysiological properties across life (Barzó et al., 2025; Guet-McCreight et al., 2023). They identified the age of the individual as a critical parameter of changes with critical changes in most of the electrophysiological parameters in the first year of life, of resting membrane potential until 40 years old and input resistance changes from this age (Barzó et al., 2025), as well as increase in sag amplitude and decrease in spike rate with age (50 years old) (Guet-McCreight et al., 2023), suggesting that reproducibility and clinical translation of drug delivery experiments needs to pay attention of these 3 periods of life (first year of life, 1–40/50 years old, >40/50 years old). Moreover, some electrophysiological properties correlates with IQ scores (Goriounova et al., 2018), suggesting that other metadata should be noted while doing such experiments.

Organotypic cortical sections were successfully cultured for a couple of weeks *ex vivo* on artificial or human cerebrospinal fluids with stable neuronal morphology and electrophysiological properties (Verwer et al., 2002; Verwer et al., 2002; Plug et al., 2024; Schwarz et al., 2019; O’Connor et al., 1997; Eugène et al., 2014; Andersson et al., 2016; Ting et al., 2018; Schwarz et al., 2017; McGeachan et al., 2025; McGeachan et al., 2025; Bak et al., 2024; Wickham et al., 2020; Andersson et al., 2016; Vormstein-Schneider et al., 2020; Chaichana et al., 2007; Jung et al., 2002; Ravi et al., 2019; Mendes et al., 2018; Sebollela et al., 2012; Barth et al., 2021; Da Seixas Silva et al., 2017; Da Seixas Silva et al., 2017; Verwer, 2003; Wu et al., 2008; Taylor et al., 2024; Le Duigou et al., 2018; McLeod et al., 2023; Andrews et al., 2020; Subramanian et al., 2017; Mukhtar et al., 2025; Chen et al., 2023; Graybuck et al., 2021; Ting et al., 2018; Schünemann et al., 2025). Such approaches enable to explore basic and pathological mechanisms in human brain tissues from the molecular to the cell to the circuit levels (Figure 2). They open the possibility to perform dynamic experiments (e.g., live imaging, calcium activity, electrophysiology) (Wickham et al., 2020; Andersson et al., 2016; Le Duigou et al., 2018; Andrews et al., 2020; Subramanian et al., 2017; Mukhtar et al., 2025) with genetic manipulations (e.g., viral injection delivery, optogenetics) (O’Connor et al., 1997; Eugène et al., 2014; Andersson et al., 2016; Ting et al., 2018; Andersson et al., 2016; Vormstein-Schneider et al., 2020; Le Duigou et al., 2018; McLeod et al., 2023; Andrews et al., 2020; Mukhtar et al., 2025; Graybuck et al., 2021; Ting et al., 2018) or drug applications (e.g., dose – response) (Ravi et al., 2019; Mendes et al., 2018; Taylor et al., 2024; Andrews et al., 2020), as well as cell grafting (Wu et al., 2008), with control conditions from the same individual. They have mostly been performed in adult conditions, non-pathological (e.g., outside an epileptic focus) (Verwer et al., 2002; Verwer et al., 2002; Schwarz et al., 2019; Andersson et al., 2016; Ting et al., 2018; Schwarz et al., 2017; Bak et al., 2024; Wickham et al., 2020; Andersson et al., 2016; Le Duigou et al., 2018; Chen et al., 2023; Graybuck et al., 2021; Ting et al., 2018; Schünemann et al., 2025) and pathological (e.g., epilepsy, tumor environment, neurodegenerative conditions) (Schwarz et al., 2019; O’Connor et al., 1997; Eugène et al., 2014; McGeachan et al., 2025; McGeachan et al., 2025; Vormstein-Schneider et al., 2020; Chaichana et al., 2007; Jung et al., 2002; Ravi et al., 2019; Mendes et al., 2018; Sebollela et al., 2012; Barth et al., 2021; Da Seixas Silva et al., 2017; Da Seixas Silva et al., 2017; Verwer, 2003; Wu et al., 2008; Taylor et al., 2024; Plug et al., 2024) with drugs or viral applications (Plug et al., 2024), but exploring neurodevelopmental stages (McLeod et al., 2023), including fetal stages (McLeod et al., 2023; Andrews et al., 2020; Subramanian et al., 2017; Mukhtar et al., 2025; Coquand et al., 2024; Coquand et al., 2021), could be expanded in the future. Moreover, genetic engineering used to label specific cell types and deliver genetic sequences on those cultures, or cell delivery approaches, should be beneficial for future therapeutic approaches in humans *in vivo*.

To conclude, *ex vivo* acute and organotypic human cultures provide beneficial approaches to understand human brain development, aging, evolution and disorders. They could provide patient-oriented therapeutical medicine, in particular for drug-resistant disorders. That is to say, the emergence of such models requires standards adopted by the community, in terms of experimental protocols (tissue transportation, cutting inhibitors, culture medium), quality assessments (electrophysiology, morphology, culture infections), metadata management (age, sex, origin, sociocultural status, IQ, etc.) and ethical standards (communication, consent approval & post-mortem donations in particular in children and intellectual deficiency conditions, genomic experiments). Another key aspect is to improve the communication between basic research, clinicians, patient involvement and companies to facilitate tissue sharing, explorative research and drug development. Moreover, preclinical drug development, whatever the preclinical model (*ex vivo*, *in vitro*, animal) requires strong relevance of the outcome and parameters assessed related to what is expected in human individuals (e.g., blood–brain barrier penetration, pharmacokinetics and dynamics, toxicity, dose selection, biomarkers, end points), to ensure higher chance of translation.

## Discussion: singularity, specificity, and experimental approaches

In this essay, I have explored multiple layers of divergence in the human lineage, from early neurodevelopment to neuronal properties and neurocognitive features. None of these differences are strictly “human-specific,” consistent with Darwin’s view that most differences between humans and other animals are “*of degree, not of kind*” (Richerson et al., 2021; Lindhout et al., 2024; Darwin, 1871). Therefore, I advocate for the concept of “species-singularity” or “human species-specificity” rather than claiming human uniqueness. The specificity of human neurodevelopment results from a complex interplay of evolutionary cellular mechanisms that influence brain cytoarchitecture, connectivity, neuronal properties, and circuit function, culminating with enhanced cognitive abilities, in a cultural species (Pollen et al., 2023). The molecular mechanisms underlying these processes are human-specific evolutionary innovations combined with hominid-, primate-, mammalian-, vertebrate-, and metazoan-conserved mechanisms (Lancaster, 2024; Tosches, 2021). Human modifiers modulate, refine or combine ancestral mechanisms in an “*evolutionary tinkering*” (Jacob, 1977).

In conclusion, while the meaning behind the stencils of human hands remains beyond our reach, experimental biology enables us to begin understanding the biological substrates of human neural evolution and of species-specific sensitivities to neurodevelopmental and neurological disorders. Complementary multimodal and multiscale approaches are beneficial to assess specificities of human neural properties, from the molecule to the behavioral levels, which could lead in the future to the discovery of novel therapeutic approaches based on human-specific cellular and molecular properties.
